# 

**Published:** 1889-10-12

**Authors:** 


					r uu. UUIL PLhi<lV.
w IX
l59, Vol. VII-
-October 12th, 188S.
? ?*? WUV/WWX JL?JUXXj XWUOI
at the General Post Office as a Newspaper.]
Per Annum, 6s. 6d., post free.
Monthly Parts, 6d.; post free 7Jd.
Ditto per Annum, 7s. 6d., post free.
'omas's Hospital
utAscofv Western Infirmary
Wcm 'folRTub Nor w/ch hospi tal
A WEEKLY INSTITUTIONAL JOURNAL OF

				

